# Pharmacological significance of heterocyclic 1*H*-benzimidazole scaffolds: a review

**DOI:** 10.1186/s13065-019-0625-4

**Published:** 2019-08-06

**Authors:** Sumit Tahlan, Sanjiv Kumar, Balasubramanian Narasimhan

**Affiliations:** Faculty of Pharmaceutical Sciences, Maharshi Dayanand University, Rohtak, 124001 India

**Keywords:** Benzimidazole derivatives, Antiprotozoal activity, Anti-inflammatory activity, Antimalarial activity, Antimycobacterial activity, Antiviral activity, Anticancer activity

## Abstract

Heterocyclic compounds are inevitable in a numerous part of life sciences. These molecules perform various noteworthy functions in nature, medication and innovation. Nitrogen-containing heterocycles exceptionally azoles family are the matter of interest in synthesis attributable to the way that they happen pervasively in pharmacologically dynamic natural products, multipurpose arranged useful materials also profoundly powerful pharmaceuticals and agrochemicals. Benzimidazole moiety is the key building block for several heterocyclic scaffolds that play central role in the biologically functioning of essential molecules. They are considered as promising class of bioactive scaffolds encompassing diverse varieties of activities like antiprotozoal, antihelminthic, antimalarial, antiviral, anti-inflammatory, antimicrobial, anti-mycobacterial and antiparasitic. Therefore in the present review we tried to compile the various pharmacological activities of different derivatives of heterocyclic benzimidazole moiety.
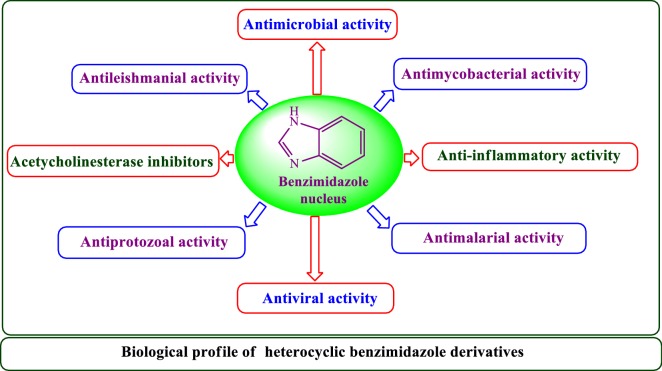

## Introduction

Among heterocyclic pharmacophores, the benzimidazole ring system is quite common. These substructures are often called ‘privileged’ due to their wide recurrence in bioactive compounds [1]. Benzimidazole moiety is a fusion of benzene and imidazole ring system at the 4 and 5 positions of imidazole ring. They have properties of both acids and bases. The NH group here is highly acidic and also feebly basic. Another feature of it is that they comprise the ability to form salts. The benzimidazole moiety is useful for the development of novel medicinal compounds in pharmaceutical field. Benzimidazole is also a vital pharmacophore, a privileged sub-structure in medicinal chemistry which contributes as a key part for different natural activities [2].

### Pharmacological significance of benzimidazole derivatives

**Fig. 1:**
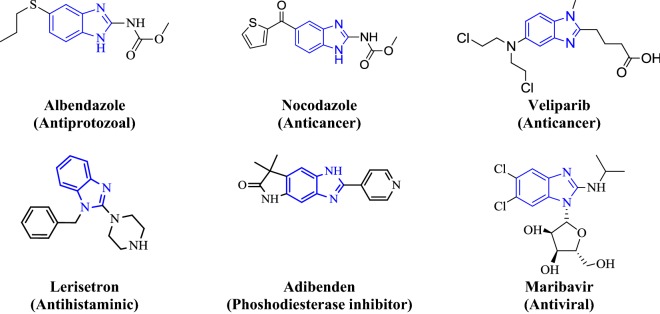
Some benzimidazoles containing medicinal preparation

Literature survey reveals that the various derivatives of benzimidazole have been synthesized for their pharmacological activities such as antimicrobial [3], anticancer [2], acetylcholinesterase [4], antiprotozoal [5], anti-inflammatory [6], analgesic [7], antihistaminic [8], antimalarial [9], antitubercular [10], anti-HIV [11] and antiviral [12]. Some of the already synthesized compounds from the above mentioned field have found very strong application in medicine praxis. The activity against bacteria, fungi and helminthes resulted their mode of action, which resulted in the blockage of microtubule in various nematode, trematode and cystode [13]. Benzimidazole-based drugs exhibit a wide range of different biological activities as a result of changing the groups on the core structure. Some marketed drugs containing benzimidazole nucleus are shown in Fig. 1.

Acetylcholinesterase (AChE) is a core chemical engaged with the ending of nerve signs via the hydrolysis of acetylcholine. It is an objective of medication advancement to battle the neuromuscular issue, for example, glaucoma, myasthenia gravis and Alzheimer’s disease (AD). AChE has been focused in the cure of AD, a dynamic neurodegenerative disease portrayed by neurofibrillary tangles, *β*-amyloid plaques and loss of focal cholinergic ability. A lack in cholinergic neurotransmission is viewed as one of the real reasons for reminiscence weaknesses in the patients with AD. One of the compelling methodologies for improving the cholinergic transmission is to utilize the inhibitors of acetylcholinesterase [4]. Parasitic ailments are as yet overall issues that deeply affect general wellbeing. Contaminations brought about by protozoa, for example, *Trypanosoma cruzi*, *Plasmodium falciparum*, *Entamoeba histolytica*, *Leishmania Mexicana*, *Trichomonas vaginalis*, *Giardia intestinalis* and helminth, for example, *Taenia solium* or *Trichinella spiralis* are overall spread ailments that influence predominantly immature nations, where tropical or template temperatures exist, yet in addition poor uncontaminated and cleanliness conditions are normal [14].

Irritation is a confined reaction of body tissues to destructive incentives or injures bringing about the arrangement of protein-rich exudates. It is a defensive reaction of the nonspecific resistant framework that expels the essential driver of cell damage; eradicate necrotic cells and tissues harmed from the incendiary procedure and commence tissue repair. The essential indications of aggravation are redness, heat, torment, swelling and loss of capacity. Reason for aggravation is physical as well chemical means, immunological responses and contamination by pathogenic life form. Aggravation can be assigned as acute and chronic. Acute irritation is described by the exudation of liquid and plasma proteins (oedema) and the development of leukocytes, particularly neutrophils. Chronic irritation is otherwise called constant aggravation, in which tissue destruction and recovering are continuing all the while, for example, tuberculosis, rheumatoid joint inflammation, constant lung infections and atherosclerosis [6].

Mosquitoes are one of the deadliest creepy crawlies in earth which generate biting irritation and also transmit lethal infections, for example, intestinal sickness, yellow fever, filariasis, chikungunya, encephalitis and dengue. Mosquitoes in the class Aedes are liable for the transmission of chikungunya, dengue, yellow fever and other pathogenic arbo-infections. Likewise, the prime vector for lymphatic filariasis is *Culex quinquefasciatus*, as well called southern house mosquito. *Cx. quinquefasciatus* ordinarily stay around human lodging and on maturing like to nibble people than different warm blooded creatures. Intestinal sickness is a mosquito-borne infectious ailment which is mostly transmitted by a contaminated female Anopheles mosquito [15].

Tuberculosis (TB), which is caused prevalently by *Mycobacterium tuberculosis* (Mtb), is the main source of death from a reparable irresistible ailment, and has been recognized by the World Health Organization (WHO) as one of the three need illnesses for medication innovative work [16]. Viral hemorrhagic fever is a genuine sickness portrayed by broad vascular harm and draining diathesis, fever and various organ inclusions. Various infections can cause this disorder, each with its very own creature repository, method of transmission, mortality rate, and clinical result in people [17].

Worldwide infectious disease figures have attained an alarming level following the proliferation of Gram-positive and Gram-negative multi-drug-resistant species. Patient non-compliance and the occurrence of multidrug-resistant pathogens often interfere innovative infection therapies that depend on a sustained multidrug course. Rational drug design has been shown to be very beneficial in this respect, since the biochemical basis of intrinsic and acquired resistance mechanisms is largely known [3].

One of the most commonly known gastrointestinal malignancies is colorectal tumor (CRC). Alterations in lifestyle, elevated-fat diet, physiological disillusionment and smoking are associated to pathogenesis of CRC. Approximately 25% of CRC cases were identified with early analysis metastases and at some stage of life nearly 50% of CRC patients would suffer from metastasis. The therapy results for these patients are largely unsatisfactory as normal regimens consider the possibility of homogeneous tumor mass distribution [2].

**Fig. 2:**
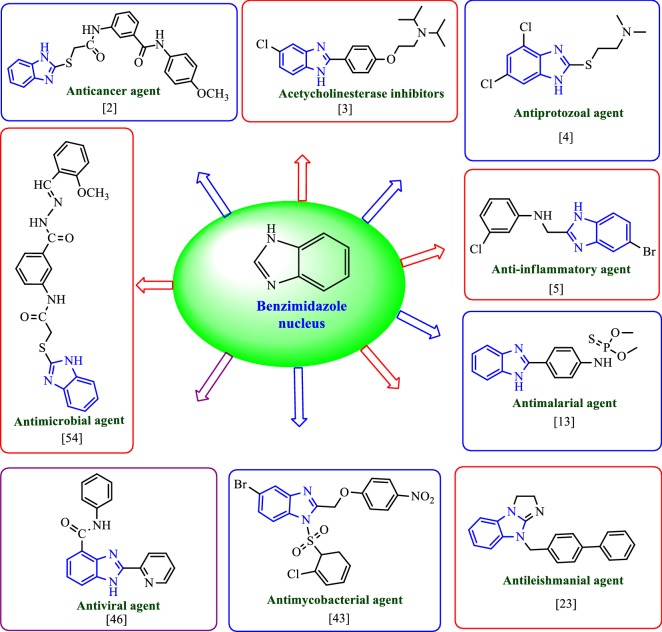
Rational designed based on literature survey of benzimidazole derivatives

Rational designed based on literature survey of benzimidazole derivatives is shown in Fig. 2.

## Reported pharmacological activities of benzimidazole derivatives

### Acetyl cholinesterase inhibitory

**Fig. 3:**
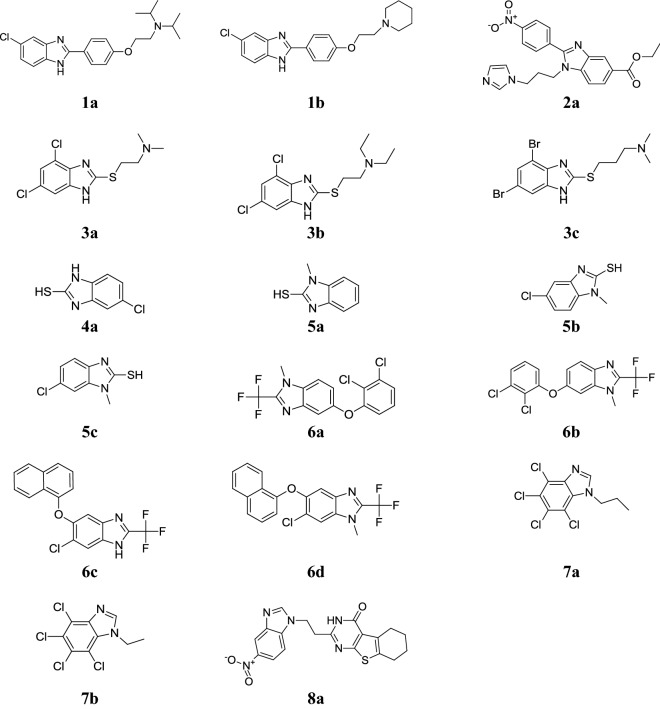
Molecular structures of compounds (**1a**–**1b**, **2a**, **3a**–**3c**, **4a**, **5a**–**5c**, **6a**–**6d**, **7a**–**7b**, **8a**)

Alpan et al. designed a class of *N*-{2-[4-(1*H*-benzimidazole-2-yl)phenoxy]ethyl} substituted amines and evaluated for its butyrylcholinesterase and acetylcholinesterase inhibitor activity. Among the synthesized derivatives, compounds **1a** and **1b** were found to be the most active against *ee*AChE and *h*AChE using tacrine as standard drug (Table 1, Fig. 3) [4].

**Table 1:** In vitro inhibition of AChE/BuChE of compounds (**1a** and **1b**)

Comp.	IC_50_ ± SEM (µM)		
	*ee*AChE	*h*AChE	BchE
**1a**	0.58 ± 0.06	3.68 ± 0.39	7.44 ± 1.51
**1b**	0.61 ± 0.07	0.13 ± 0.03	> 100
**Tacrine**	0.075 ± 0.02	0.52 ± 0.09	0.0098 ± 0.0002

Yoon et al. synthesized a class of benzimidazoles and screened for its acetylcholinesterase and butyrylcholinesterase inhibitor activity. Compound **2a** (Fig. 3) showed promising inhibitory activity with IC_50_ = 5.12 µM for BChE and IC_50_ = 8.63 µM for AChE using rivastigmine and donepezil (22.00, 7.95 µM for BChE and 50.20, 0.03 µM for AChE) as standard [18].

### Antiprotozoal activity

Andrzejewska et al. synthesized two series of *S*-substituted 4,6-dihalogeno-2-mercapto-1*H*-benzimidazoles and assessed for their in vitro antiprotozoal potential towards *G. intestinalis* and *T. vaginalis* using albendazole and metronidazole as standard. Among them, compounds **3a**, **3b** and **3c** were found to be most potent and comparable to standard drugs (Table 2, Fig. 3) [5].

**Table 2:** Antiprotozoal activity of benzimidazole compounds (**3a**–**3c**)

Comp.	IC_50_ µg/mL	
	*Giardia intestinalis*	*Trichomonas vaginalis*
**3a**	0.006	0.021
**3b**	0.006	0.013
**3c**	0.008	0.004
**Albendazole**	0.010	0.422
**Metronidazole**	0.210	0.037

Diaz-Chiguer et al. prepared a new series of benzimidazole derivatives and evaluated in vitro (via the % of lysis of bloodstream) and in vivo for its trypanocidal activity against of *Trypanosoma cruzi* (NINOA and INC5). In this series, compound **4a** showed significant in vitro and in vivo [INC5: 68.4 (% lysis); NINOA: 46.4 (% lysis)] trypanocidal activity (Table 3, Fig. 3) [19].

**Table 3:** In vitro trypanocidal activity of synthesized compound **4a**

Comp.	*Trypanosoma cruzi*				
	LC_50_ (mM)		CC_50_ (mM)	Selectivity index (SI)	
	INC5	NINOA		INC5	NINOA
**4a**	0.32	0.014	43.2	135	3085.71
**Nifurtimox**	0.69	0.78	25.4	36.81	32.56
**Benznidazole**	0.31	0.60	23.6	76.13	39.3

Hernandez-Covarrubias et al. reported a class of benzimidazoles and evaluated for its antiprotozoal activity against *G. duodenalis*. All the tested compounds were found to be more active than standard metronidazole but the better activity observed with SH group compounds **5a**–**5c** (Fig. 3) (IC_50_ = 18–45 µM) which exhibited considerable activity as compared to metronidazole (IC_50_ = 1.22 µM) [20].

Hernandez-Luis et al. synthesized a series of 2-(trifluoromethyl)-1*H*-benzimidazole molecules and assessed in vitro for its antiparasitic activity towards various protozoan parasites: *G. intestinalis* (GI), *T. vaginalis* (TV) *E. histolytica* (EH) and *L. mexicana* (LM) using albendazole (ABZ), mebendazole (MBZ), pentamidine as standard drugs and in vivo towards *Trichinella spiralis* (TS) using albendazole (ABZ), triclabendazole (TBZ) and pentamidine as standard drugs. In this class, compounds **6a**, **6b** and **6c** exhibited good antiparasitic activity and in addition, compound **6a** and **6c** showed good activity against *T. spiralis* at adult phase and **6d** possessed the good antiprotozoal potential against the muscle larvae stage (Tables 4 and 5, Fig. 3) [14].

**Table 4:** In vitro antiprotozoal and anthelmintic screening results

Comp.	Microbial strains (IC_50_ µg/mL)						
	GI	EH	TV	LM	TS (% reduction, 0.18 µM)	TS (% reduction, 0.37 µM)	TS (% reduction, 1.80 µM)
**6a**	0.030	0.009	0.016	24.00	54 ± 2	62 ± 2	80 ± 3
**6b**	0.063	0.019	0.110	4.10	44 ± 2	48 ± 3	67 ± 2
**6c**	0.005	0.019	0.086	13.78	43 ± 3	50 ± 2	65 ± 3
**ABZ**	0.037	56.6	1.592	^a^	58.6 ± 2	61.9 ± 3	67 ± 6
**MTZ**	1.228	0.350	0.216	^b^	^b^	^b^	^b^
**Pentamidine**	^b^	^b^	^b^	2.421	^b^	^b^	^b^

^a^No effect

^b^Not determined

**Table 5:** Percentage of adult and muscle larvae load reduction in *T. spiralis*

Comp.	Adult phase	Muscle larvae stage	
	50 mg/kg	75 mg/kg	75 mg/kg
**6a**	^a^	58	46
**6c**	69	80	40
**6d**	^b^	36	64
**ABZ**	62	73	63
**MTZ**	41	7	25
**Alpha**	28	^a^	24
**Control**	0	0	0

^a^Not determined

^b^No reduction

Kopanska et al. reported a series of 1*H*-benzimidazole analogues and assessed for its in vitro antiprotozoal activity against *Acanthamoeba castellanii* and compared with chlorhexidine as reference. The screening results indicated that compounds **7a** and **7b** were found most efficient in reducing the figure of trophozoites and cysts (Table 6, Fig. 3) [21].

**Table 6:** Reduction in viability of *A. castellanii* trophozoites and cysts

Comp.	Concentrations [µmol/L]	% of survivors			Percentage content of particular stages	
		Trophozoites	Cysts	Total	Trophozoites	Cysts
**7a**	5.5	23.3 ± 2.0	15.0 ± 2.3	22.5 ± 2.0	93.4 ± 8.0	6.6 ± 1.0
	11.1	41.2 ± 2.8	76.0 ± 9.7	44.5 ± 3.5	83.6 ± 5.7	16.4 ± 2.1
**7b**	5.2	26.5 ± 2.3	19.0 ± 3.4	25.8 ± 2.4	92.7 ± 7.9	7.3 ± 1.3
	7.9	22.0 ± 1.8	121.0 ± 12.6	31.6 ± 2.9	62.5 ± 5.2	37.5 ± 3.9
**Chlorohexidine**	4.4	23.4 ± 0.7	11.0 ± 1.6	22.3 ± 0.8	95.3 ± 2.9	4.7 ± 0.7
	11.0	24.2 ± 1.1	31.0 ± 4.8	24.8 ± 2.6	88.4 ± 3.9	11.6 ± 1.8

Mavrova et al. synthesized novel derivatives of thieno[2,3-*d*]pyrimidin-4(3*H*)-ones and screened for their in vitro antiparasitic activity against *Trichinella spiralis* using albendazole (as standard drug). Among them, compound **8a** showed good antiparasitic activity. The significance results of the active compound shown in Table 7 and Fig. 3 [22].

**Table 7:** Antihelmintic activity of compound 8a against *Trichinella spiralis*

Comp.	Efficacy (%) after 24 h	Efficacy (%) after 48 h
**8a**	95.05	95.05
**Albendazole**	10.6	14.8

**Fig. 4:**
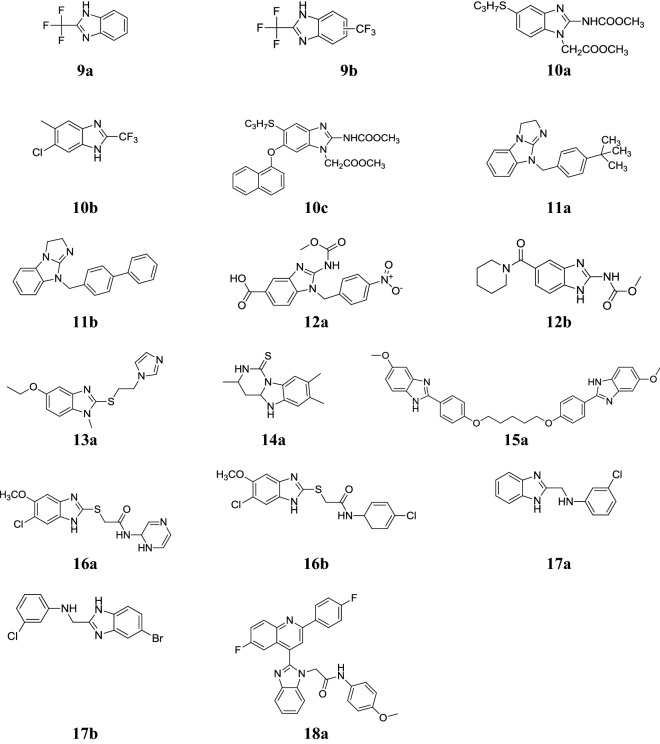
Molecular structures of compounds (**9a**–**9b**, **10a**–**10c**, **11a**–**11b**, **12a**–**12b**, **13a**, **14a**, **15a**, **16a**–**16b**, **17a**–**17b**, **18a**)

Navarrete-Vazquez et al. synthesized a sequence of 2-(trifluoromethyl)-1*H*-benzimidazoles along with various bioisosteric substituents at 5- and 6-position (–Cl, –F, –CF_3_, –CN) and examined for its in vitro antiprotozoal activity towards the protozoa *T. vaginalis* and *G. intestinalis* using metronidazole and albendazole as reference. In this series, compound **9a** showed most promising activity than metronidazole against *G. intestinalis* and compound **9b** found more active against *T. vaginalis* than the reference drugs. The compound **9b** as well displayed modest antimalarial activity against D6 and W2 strains of *Plasmodium falciparum* (Table 8, Fig. 4) [23].

**Table 8:** IC_50_ (μM) of synthesized compounds (**9a** and **9b**)

Comp.	*G. intestinalis*	*T. vaginalis*	*P. falciparum*	
			D6	W2
**9a**	0.107 ± 0.017	3.314 ± 0.130	> 20	> 20
**9b**	0.672 ± 0.020	0.232 ± 0.021	5.98 ± 0.25	6.12 ± 0.32
**Metronidazole**	1.226 ± 0.125	0.236 ± 0.016	–	–
**Albendazole**	0.038 ± 0.003	3.390 ± 0.125	> 20	>20

Marquez-Navarro et al. developed new derivatives of benzimidazole moiety and examined for their in vivo antiprotozoal activity toward *Hymenolepis nana* adult and in vitro toward *Toxocara canis* larvae. In vitro screening results indicated that compound **10a** showed significant activity toward *T. canis* whereas compounds **10b** and **10c** showed the good in vivo results against *H. nana* and compared to standard albendazole (Table 9, Fig. 4) [24].

**Table 9:** Antihelmintic screening results

Comp.	C log P	*T. canis* J2 larvae in vitro relative mobility (%)			*H. nana* in vivo adult reduction (%)
		0.18 µM	1.8 µM	18 µM	50 mg/kg
**10a**	3.23	40	40	30	–
**10b**	–	–	–	–	97
**10c**	–	–	–	–	96
**Albendazole**	3.01	80	40	40	83

Oh et al. synthesized a novel class of 2,3-dihydroimidazo[1,2-*a*]benzimidazole and screened for its anti-leishmanial and anti-trypanosomal activities towards *Leishmania donovani* and *Trypanosoma cruzi* using miltefosine, benznidazole and amphotericin B as standard. Compounds **11a** and **11b** showed promising antiprotozoal activity (Tables 10 and 11, Fig. 4) [25].

**Table 10:** In vitro anti-leishmanial screening results

Comp.	*Leishmania donovani*			
	Amastigote form			Promastigote form
	EC_50_ (μM)	CC_50_ (μM)	SI	EC_50_ (μM)
**11a**	3.05	> 50	> 16.4	1.25
**11b**	5.29	39.7	7.5	1.48
**Miltefosine**	4.83	18.9	3.91	11.1
**Amphotericin B**	0.25	7.57	30.2	0.22

*CC*_*50*_ cytotoxicity, *EC*_*50*_ half maximal effective concentration, *SI* selective index (EC_50_/CC_50_)

**Table 11:** In vitro anti-trypanosomal screening results

Comp.	*Trypanosoma cruzi*		
	EC_50_ (μM)	CC_50_ (μM)	SI
**11a**	1.10	36.5	33.2
**11b**	2.10	18.8	8.95
**Benznidazole**	20.7	> 50	> 2.42

Palomares-Alonso et al. developed new substituted benzimidazoles and assessed for their cysticidal activity against *Taenia crassiceps* cysts (ORF and WFU strain) using albendazole sulfoxide as control drug. Among them, compounds **12a** and **12b** displayed superior cysticidal activity (Table 12, Fig. 4) [26].

**Table 12:** Cysticidal activity against *T. crassiceps* (ORF and WFU strains)

Comp.	Cysts mortality (%)			
	ORF strain		WFU strain	
	0.28 µM	1.70 µM	0.28 µM	1.70 µM
**12a**	41 ± 4.6	68 ± 7	22.6 ± 2.3	26 ± 4
**12b**	37 ± 6.1	62 ± 8	6.3 ± 2.3	16.7 ± 3
**Albendazole sulfoxide**	46 ± 5	88 ± 7	25 ± 2.3	35 ± 2.3

Perez-Villanueva et al. synthesized a new class of 2-{[2-(1*H*-imidazol-1-yl)ethyl]-sulfanyl}-1*H*-benzimidazole derivatives and assessed for its in vitro antiprotozoal activity against protozoa *G. intestinalis, T. vaginalis* and *E. histolytica* using metronidazole and albendazole as standard drugs. Among them, compound **13a** showed highest activity against *G. intestinalis* (Table 13, Fig. 4) [27].

**Table 13:** Antiprotozoal screening results

Comp.	Microbial strains IC_50_ (µM)		
	*T. vaginalis*	*G. intestinalis*	*E. histolytica*
**13a**	0.0761 ± 0.0094	0.0083 ± 0.0023	0.0298 ± 0.0047
**Meteronidazole**	0.2360 ± 0.0160	1.2260 ± 0.1250	0.3798 ± 0.1461
**Albendazole**	1.5905 ± 0.0113	0.0370 ± 0.0030	56.5334 ± 18.8445

Sondhi et al. synthesized pyrimido[1,6-*a*]benzimidazoles and assessed for their in vitro antiamoebic activity by microdilution method against *E. histolytica*. In this series, compound **14a** (Fig. 4) showed best IC_50_ value 1.82 µM as compared to metronidazole which showed IC_50_ value 1.22 µM [28].

Torres-Gomez et al. reported some benzimidazole pentamidine compounds and assessed for their in vitro antiprotozoal activity against *L. Mexicana*, *E. histolytica*, *Giardia lamblia*, *T. vaginalis* and *Plasmodium berghei* using pentamidine and metronidazole (as reference drugs). Among the reported compounds, compound **15a** showed good activity against *G. lamblia*, *E. histolytica, L. mexicana* and *T. vaginalis* and comparable to standard pentamidine (Table 14, Fig. 4) [29].

**Table 14:** Antiprotozoal screening results

Comp.	Microbial strains (IC_50_ µ/M)				
	*T. vaginalis*	*G. lamblia*	*E. histolytica*	*L. mexicana*	*P. berghei*
**15a**	0.164	0.435	0.109	34.641	0.712
**Pentamidine**	3.815	4.079	11.801	^a^	9.568
**Meteronidazole**	0.286	1.286	0.771	–	–

– Not tested

^a^Cell damage, due to cytophatic effect caused by pentamidine

Velazquez-Lopez et al. reported some new benzimidazole derivatives and evaluated for their in vitro antiprotozoal activity against *T. cruzi* epimastigotes INC-5 and NINOA using reference drug (nifurtimox). Among the synthesized compounds, compound **16a** showed potent activity towards the *T. cruzi* epimastigote INC-5 strain while compound **16b** found active against the NINOA strain and comparable to nifurtimox (Table 15, Fig. 4) [30].

**Table 15:** In vitro susceptibility of bloodstream epimastigote

Comp.	IC_50_ INC-5 (μM)	IC_50_ NINOA (μM)	CC_50_ (μM)
**16a**	28.672 ± 0.602	98.799 ± 1.990	134.580 ± 1.995
**16b**	186.230 ± 4.103	56.967 ± 0.961	90.436 ± 1.426
**Nifurtimox**	50.750 ± 0.839	89.804 ± 1.138	131.503 ± 0.490

### Anti-inflammatory activity

Achar et al. prepared a class of 2-methylaminobenzimidazole compounds and screened in vivo for its analgesic (acetic acid induced writhing in mice) and anti-inflammatory activities (carrageenan induced paw oedema in rats). Among them, compounds **17a** and **17b** were displayed considerable analgesic and anti-inflammatory activities in comparison to reference nimesulide (Tables 16, 17 and 18, Fig. 4) [6].

**Table 16:** Analgesic screening results

Comp.	Mean values (X ± SE)	(%) Protection
**Control**	300 ± 1.55	–
**17a**	5.6 ± 1.85	81.33
**17b**	3.3 ± 1.66	89.00
**Nimesulide**	–	100

**Table 17:** Anti-inflammatory screening results

Comp.	Paw oedema thickness (mm)			
	30 m (X ± SE)	Oedema Inhibition (%)	60 m (X ± SE)	Oedema Inhibition (%)
**Control**	1.3 ± 0.05	–	1.5 ± 0.03	–
**17a**	1.1 ± 0.03	15.3	1.1 ± 0.00	26.6
**17b**	1.2 ± 0.03	7.6	1.1 ± 0.06	26.6
**Nimesulide**	1.1 ± 0.05	15.3	1.1 ± 0.00	26.6

**Table 18:** Anti-inflammatory screening results

Comp.	Paw oedema thickness (mm)			
	120 m (X ± SE)	Oedema Inhibition (%)	180 m (X ± SE)	Oedema Inhibition (%)
**Control**	1.7 ± 0.03	–	1.8 ± 0.03	–
**17a**	1.1 ± 0.03	41.1	1.0 ± 0.03	44.4
**17b**	1.4 ± 0.03	17.6	1.5 ± 0.05	16.6
**Nimesulide**	1.0 ± 0.00	41.1	1.1 ± 0.00	44.4

El-Feky et al. designed novel fluorinated quinoline incorporated benzimidazoles and evaluated for their in vivo anti-inflammatory activity by carrageenin induced edema bioassay method in rats using celecoxib. Among them, compound **18a** demonstrated the highest anti-inflammatory activity and exhibited best binding profiles into the COX-2 binding site as compared to celecoxib. The significance result of the active compound is shown in Table 19, Fig. 4 [31].

**Table 19:** Anti-inflammatory screening results

Comp	Anti-inflammatory activity
	Protection at 50 mg/kg dose (%)
**18a**	55
**Celecoxib**	50

**Fig. 5:**
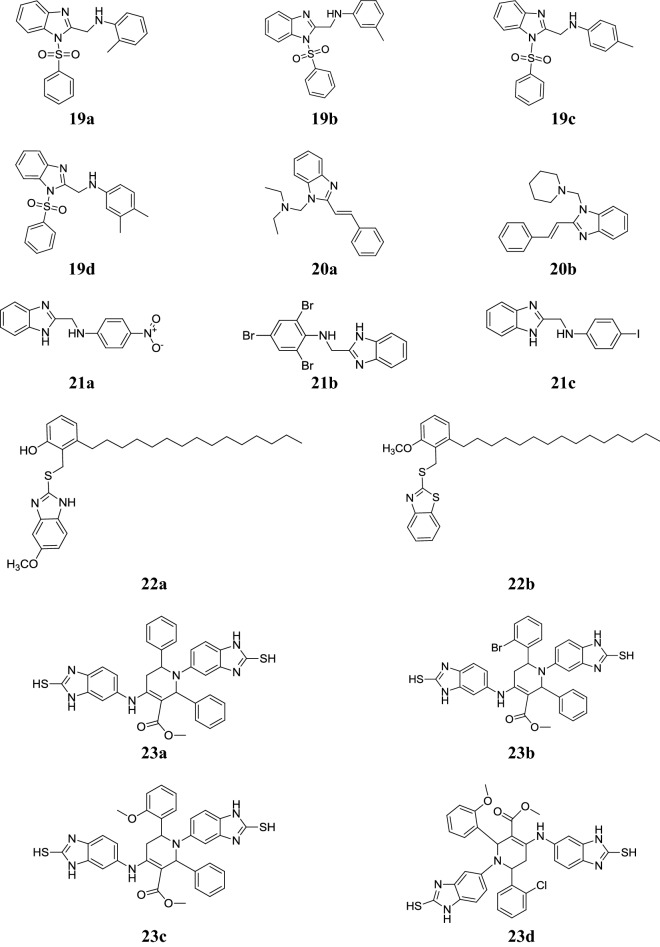
Molecular structures of compounds (**19a**–**19d**, **20a**–**20b**, **21a**–**21c**, **22a**–**22b**, **23a**–**23d**)

Gaba et al. reported phenylsulfonyl substituted benzimidazoles and evaluated in vivo for their anti-inflammatory activity (carrageenan-induced paw edema in rats) and analgesic activity (acetic acid-induced writhing test in mice), respectively. Among them, compounds **19a**, **19b**, **19c** and **19d** showed significant reduction in edema and compared to standard drug indomethacin and protection in the number of writhes produced by acetic acid, and comparable to the reference drug acetyl salicylic acid (Tables 20 and 21, Fig. 5) [7].

**Table 20:** Anti-inflammatory screening results

Comp.	Edema at 3 h (%, mean ± SEM)	Reduction in edema (%)
**19a**	68.66 ± 72.99	31.34
**19b**	67.16 ± 73.06	32.84
**19c**	65.67 ± 73.78	34.33
**19d**	62.69 ± 73.27	37.31
**Control**	100.00 ± 73.59	0.00
**Indomethacin**	52.23 ± 74.27	47.76

**Table 21:** Analgesic screening results

Comp.	No. of writhes in 15 min (%, Mean ± SEM)	Protection (%)
**19a**	32.33 ± 73.62	54.03
**19b**	33.17 ± 73.39	52.84
**19c**	32.67 ± 73.57	53.55
**19d**	29.83 ± 72.45	57.58
**Control**	70.33 ± 73.01	0.00
**Acetyl salicylic acid**	25.67 ± 71.45 6	3.51

Jesudason et al. reported a class of *N*-Mannich bases of benzimidazole compounds and screened for its analgesic activity by the acetic acid induced writhing method using Wistar albino mice and anti-inflammatory activity by the formalin-induced paw edema method on Wistar albino rats by plethysmography. In this series, compound **20a** exhibited similar results to paracetamol and compound **20b** showed more potent than diclofenac (Tables 22 and 23, Fig. 5) [32].

**Table 22:** Analgesic screening results and LD_50_

Comp.	Dose (mg/kg)	% Protection	LD_50_ (mg/kg)
**20a**	20	32.2	175
	40	47.49	
**Paracetamol**	100	47.76	–

**Table 23:** Anti-inflammatory screening results

Comp.	Dose (mg/kg)	% Reduction of edema			
		30 min	60 min	90 min	120 min
**20b**	40	48	56	59	62
**Diclofenac**	50	48	65	64	65

Mariappan et al. developed some 2-substituted benzimidazole molecules and screened for their in vivo anti-inflammatory and analgesic activities using pentazocine as standard. Among the synthesized derivatives, compounds **21a**, **21b**, **21c** showed significant analgesic and anti-inflammatory activity (Tables 24 and 25, Fig. 5) [33].

**Table 24:** Analgesic activities of benzimidazole compounds 21a-c via Tail-flick method

Comp.	(Mean ± SEM) tail withdrawing time in second				
	0 h	1 h	2 h	3 h	4 h
**Control (0.5% CMC)**	1.56 ± 0.16	2.16 ± 0.16	2.33 ± 0.21	2.66 ± 0.21	2.82 ± 0.72
**Pentazocine**	2.16 ± 0.16	8.5 ± 0.34	11.33 ± 0.21	10.16 ± 0.30	10.83 ± 0.30
**21a**	2.0 ± 0.25	3.0 ± 0.25	4.16 ± 0.33	10.5 ± 0.22	9.83 ± 0.33
**21b**	2.0 ± 0.25	4.33 ± 0.21	3.73 ± 0.30	8.63 ± 0.21	10.03 ± 0.30
**21c**	2.0 ± 0.25	6.51 ± 0.21	7.83 ± 0.30	9.73 ± 0.21	9.25 ± 0.30

**Table 25:** Anti-inflammatory activities of benzimidazole compounds 21a-c by carrageenan-induced rat paws edema method

Comp.	(Mean ± SEM) tail withdrawing time in second					Inhibition (%)
	0 h	1 h	2 h	3 h	4 h	4 h
**Control (0.5% CMC)**	0.14 ± 0.01	0.23 ± 0.01	0.24 ± 0.02	0.25 ± 0.01	0.25 ± 0.01	–
**Pentazocine**	0.14 ± 0.01	0.12 ± 0.01	0.12 ± 0.01	0.10 ± 0.01	0.09 ± 0.01	64
**21a**	0.14 ± 0.02	0.12 ± 0.02	0.11 ± 0.01	0.11 ± 0.02	0.10 ± 0.01	60
**21b**	0.15 ± 0.02	0.15 ± 0.01	0.13 ± 0.01	0.13 ± 0.01	0.10 ± 0.01	60
**21c**	0.14 ± 0.01	0.13 ± 0.01	0.12 ± 0.01	0.10 ± 0.11	0.09 ± 0.02	64

Paramashivappa et al. synthesized a class of substituted benzimidazoles and assessed for its human cyclooxygenase-2 (COX-2) and cyclooxygenase-1 (COX-1) enzyme inhibition activity in human whole blood assay using rofecoxib as reference. In this series, compound **22a** and **22b** were found as most active agents (Table 26, Fig. 5) [34].

**Table 26:** Inhibitory effect on COX-2 and COX-1 activity in human whole blood assay

Comp.	COX-2 IC_50_ µM	COX-1 IC_50_ µM	COX-1/COX-2
**22a**	1	384	384
**22b**	1.06	> 500	> 470
**Rofecoxib**	0.057	11.4	200

Ravindernath et al. designed new benzo[*d*]imidazolyl tetrahydropyridine carboxylates and evaluated for their anti-inflammatory activity by the Carrageenan-induced paw edema test in rats using diclofenac sodium as a reference drug for comparison. All synthesized compounds (**23a**–**23d**) displayed appreciable activity. The significance results of the active compounds are shown in Table 27, Fig. 5 [35].

**Table 27:** Anti-inflammatory screening results

Comp.	Time			
	1 h	2 h	3 h	4 h
**23a**	0.78 ± 0.022	1.45 ± 0.057	0.5 ± 0.027	0.08 ± 0.003
**23b**	0.55 ± 0.0389	1.583 ± 0.045	0.616 ± 0.0315	0.3 ± 0.023
**23c**	0.64 ± 0.011	1.4 ± 0.038	0.31 ± 0.024	0.31 ± 0.024
**23d**	0.82 ± 0.030	1.76 ± 0.07	0.58 ± 0.03	0.1 ± 0.002
**Control**	0.90 ± 0.04	1.60 ± 0.018	2.38 ± 0.02	3.25 ± 0.03
**Diclofenac sodium**	0.95 ± 0.03	1.72 ± 0.03	0.60 ± 0.03	0.60 ± 0.02

**Fig. 6:**
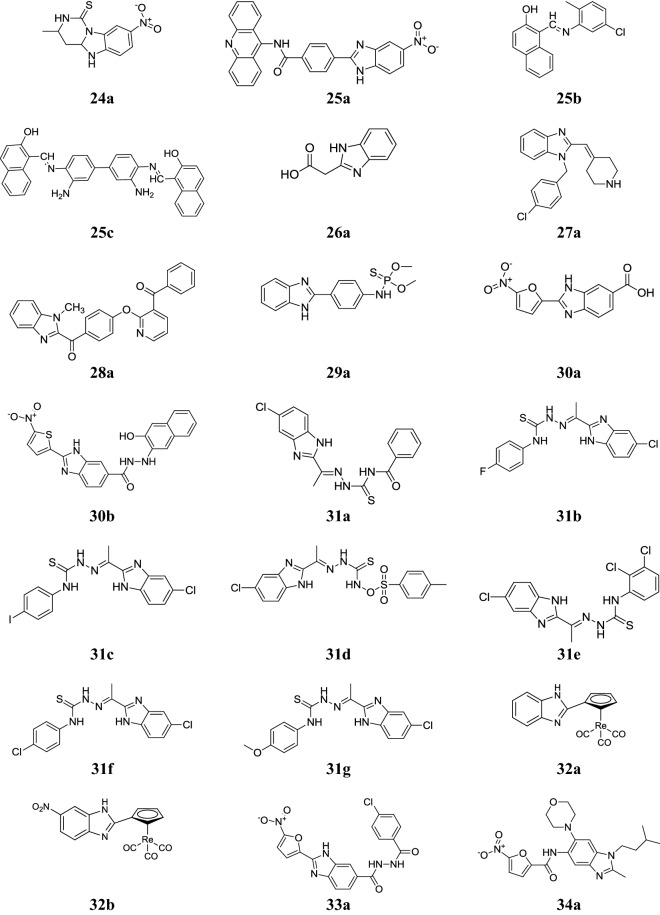
Molecular structures of compounds (**24a**, **25a**–**25c**, **26a**, **27a**, **28a**, **29a**, **30a**–**30b**, **31a**–**31g**, **32a**–**32b**, **33a** and **34a**)

Sondhi et al. synthesized pyrimido[1,6-*a*]benzimidazoles and tested in vitro for their anti-inflammatory and analgesic activities using carrageenin induced paw oedema model. Among the synthesized compounds, compound **24a** (Fig. 6) displayed superior anti-inflammatory (46%) and mild analgesic activity (50%) using ibuprofen as standard (51% and 75%) [28].

Sondhi et al. developed a class of benzimidazole acridine derivatives and tested for its anti-inflammatory, analgesic and kinase (CDK-1, CDK-5 and GSK-3) inhibition activities using ibuprofen as standard. Among the series, compound **25a** displayed considerable activity against kinase while compounds **25b** and **25c** displayed significant anti-inflammatory and analgesic activities (Table 28, Fig. 6) [36].

**Table 28:** Anti-inflammatory, analgesic and kinase inhibition activities

Comp.	Anti-inflammatory activity (%)	Analgesic activity (%)	Kinase IC_50_ (µM)		
			CDK-1	CDK-5	GSK-3
**25a**	–	–	7.4	4.6	42
**25b**	31.4	60	–	–	–
**25c**	35.8	50	–	–	–
**Ibuprofen**	38.8	50	–	–	–

Vicini et al. synthesized benzimidazole tetrazolyl- and carboxyl-derivatives and screened for their anti-inflammatory and antipyretic activities in rat paw oedema and rat *Escherichia coli* derived LPS-induced pyrexia along with antinociceptive property examined in writhing and hot plate tests in mice. Among them, compound **26a** (1*H*-benzimidazol-2-yl) acetic acid showed central analgesic activity. The significance results of the active compounds are shown in Table 29, Fig. 6 [37].

**Table 29:** Analgesic effect of compound 26a against acetic acid induced writhing in mice

Comp.	ID_50_ (mg/kg os)	Maximal inhibition % mean ± SEM
**26a**	> 200	42 ± 15
**Acetaminophen**	208	90 ± 17

Wang et al. prepared a class of benzimidazole compounds and assessed for its in vitro H_1_ antihistamine activity. Among them, compound **27a** found to display excellent activity to reduce mast cell degranulation, moderate anti-PAF activity and decreased potency on hERG as compared to standard astermizole and desloratadine (Table 30, Fig. 6) [8].

**Table 30:** Antihistamine, receptor binding and anti-PAF activities

Comp.	Anti H_1_ activity ileum IC_50_ (µmol/L)	H_1_ receptor binding IC_50_ (µmol/L)	PAF-induced platelet Aggregation IC_50_ (µmol/L)
**27a**	0.00794	0.000881	78
**Desloratadine**	0.0721	0.00588	130
**Astermizole**	0.0453	0.004	ND

Yang et al. designed new benzimidazoles and then assessed for their in vitro phosphodiesterase 10A (PDE10A) inhibitor activity. From the newly developed compounds, compound **28a** (Fig. 6) showed good IC_50_ = 3.73 ± 0.60 nM along with selectivity (> 1000-fold) for PDE10A [38].

### Antimalarial activity

Bandyopadhyay et al. synthesized new thiophosphorylated and phosphorylated benzimidazole derivatives and examined for their antimalarial activity toward *Aedes albopictus* and *Culex quinquefasciatus* for mosquito larvicidal properties at different concentration. Compound **29a** (Fig. 6) found most active toward *Ae. albopictus* (LC_50_—6.42 and 5.25 mg/L at 24 and 48 h) and *Cx. Quinquefasciatus* (LC_50_—7.01 and 3.88 mg/L) using temephos as positive control (2.85 ± 2.64, 2.8 ± 3.04 toward *Ae. Albopictus* and for *Cx. Quinquefasciatus* 3.04 ± 2.31, 3.55 ± 2.45) [15].

Camacho et al. synthesized a class of *N*′-substituted-2-(5-nitrofuran or 5-nitrothiophen-2-yl)-3*H*-benzo[*d*]imidazole-5-carbohydrazides and investigated in vitro for its efficiency to inhibit *β*-hematin formation (I*β*HS), hemoglobin hydrolysis and then in vivo in rodent *Plasmodium berghei* for its antimalarial efficacy. Compounds **30a** and **30b** showed good antimalarial activity via inhibition of *β*-hematin formation and as proficient as chloroquine (Table 31, Fig. 6) [9].

**Table 31:** Antimalarial activity of benzimidazole derivatives (30a and 30b)

Comp.	I*β*HS	IC_50_ (µM)	IGP	% P	SD
**30a**	95.43 ± 0.58	8.43	0	4.02 ± 0.45	17 ± 1.26
**30b**	75.76 ± 0.99	11.10	14.08 ± 0.88	1.8 ± 0.49	18.8 ± 2.05
**Leupeptin**	–	–	91.62 ± 0.69	–	–
**Pepstatin**	–	–	95.45 ± 0.66	–	–
**Chloroquine**	94.19 ± 0.36	–	24.12 ± 1.16	1.3 ± 0.3	> 30
**Saline Solution**	–	–	–	21.8 ± 2.31	11.66 ± 1.66

Divatia et al. synthesized novel thiosemicarbazones containing benzimidazole nucleus and evaluated for their in vitro antimalarial activity towards *P. falciparum* by minimum inhibitory concentration using chloroquine and quinine as standards. Among them, compounds **31a**, **31b**, **31c**, **31d**, **31e**, **31f** and **31g** showed excellent antimalarial activity. From structure activity relationship study it was observed that compounds having electron withdrawing groups (EWG) (*chloro*, *fluoro* and *iodo*) showed promising activity (Table 32, Fig. 6) [39].

**Table 32:** Antimalarial activity of benzimidazole derivatives 30a and 31 g

Comp.	Minimum inhibitory concentration (IC_50_ µg/mL)
**31a**	0.023
**31b**	0.003
**31c**	0.012
**31d**	0.025
**31e**	0.005
**31f**	0.26
**31** **g**	0.15
**Quinine**	0.268
**Chloroquine**	0.020

Toro et al. reported ferrocenyl and cyrhetrenyl benzimidazoles and evaluated for their in vitro antimalarial activity against chloroquine susceptible-strain (3D7) and the chloroquine resistant-strain (W2) of *Plasmodium falciparum*. A better activity was observed for the compounds **32a–32b** (Fig. 6) (IC_50_ = 10.4–26.5 μM) than its ferrocenyl analog 1-Fe-(H, NO_2_) (IC_50_ = 23.9–48.0 μM) [40].

### Anti-mycobacterial/antitubercular activity

Camacho et al. synthesized some novel *N*′-substituted-2-(5-nitrofuran or 5-nitrothiophen-2-yl)-3*H*-benzo[*d*]imidazole-5-carbohydrazide compounds and investigated for their antitubercular potency against multidrug resistant MDR-MTB and MTB H_37_Rv strains. Compounds **33a** (Fig. 6) exhibited good anti-mycobacterial activity (MIC = 12.5 µg/mL) against sensitive *M. tuberculosis* H_37_Rv and MDR-MTB (MIC = 6.25 µg/mL) strains and compared to isoniazid (MIC = 0.063 µg/mL) and rifampin (MIC = 32 µg/mL) [9].

Gong et al. reported a new series of substituted benzimidazole derivatives and investigated for their antitubercular potency against *M. tuberculosis* in a replicating state (R-*Mtb*), a physiologically-induced non-replicating state (NR-*Mtb*). Among the synthesized derivatives, compound **34a** (Fig. 6) (NR-*Mtb*: MIC_90_ = 0.20 µg/mL; R-*Mtb*: MIC_90_ < 0.049 µg/mL) [16].

**Fig. 7:**
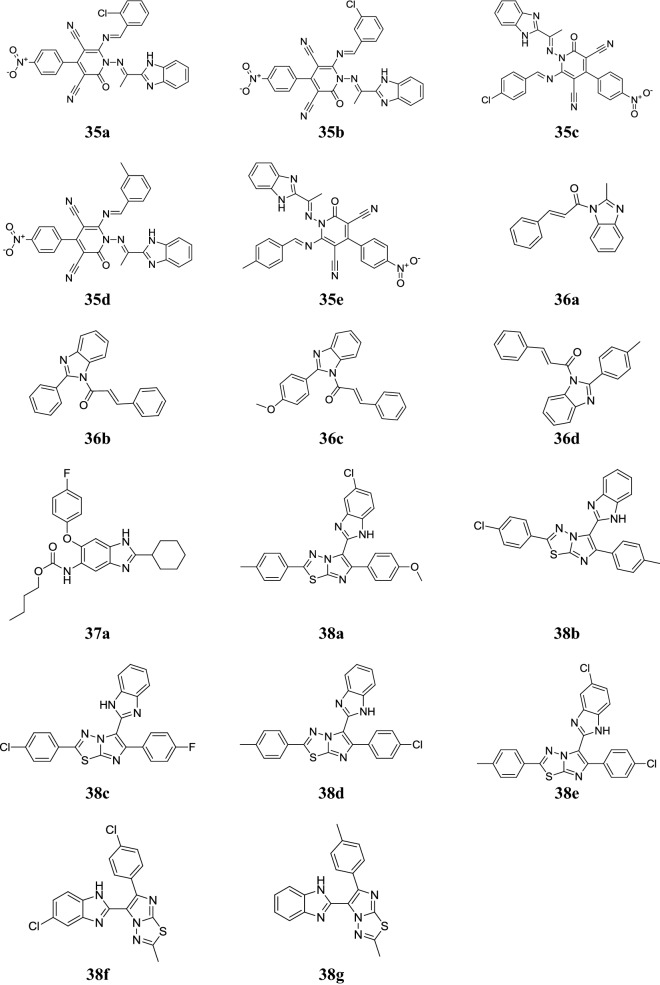
Molecular structures of compounds (**35a**–**35e**, **36a**–**36d**, **37a**, **38a**–**38g**)

Desai et al. reported a class of benzimidazole containing 2-pyridones compounds and evaluated for its antimycobacterial potential towards *M. tuberculosis* H_37_Rv strain in Middlebrook 7H12 medium by microplate alamar blue assay (MABA) using isoniazid as a reference drug. In this series, compounds **35a**, **35b**, **35c**, **35d** and **35e** (Fig. 7) exhibited good activity with MIC values (2.76–20.4 µM) as compared to isoniazid with MIC value (0.24 µM) [10].

Kalalbandi et al. developed a novel class of 1-[(2*E*)-3-phenylprop-2-enoyl]-1*H*-benzimidazoles and assessed for its antitubercular activity towards *M. tuberculosis* H_37_Rv by microplate alamar blue assay. Among them, compounds **36a**, **36b**, **36c** and **36d** (Fig. 7) (MIC = 3.12, 6.25, 3.12 and 1.6 µg/mL, respectively) showed excellent in vitro activity against H_37_Rv strain as compared to pyrazinamide, streptomycin and rifampicin having MIC = 3.12, 6.25 and 0.12 µg/mL, respectively [41].

Park et al. synthesized some new 2,5,6-trisubstituted benzimidazoles and assessed for their antitubercular potential against drug sensitive *Mtb* H_37_Rv strain using microplate alamar blue assay. Compound **37a** (Fig. 7) displayed the best potency with the MIC value (0.63 µg/mL) against *Mtb* H_37_Rv [42].

Ramprasad et al. synthesized some imidazo[2,1-*b*] [1, 3, 4] thiadiazole-benzimidazole compounds and evaluated for their in vitro antituberculosis potency against *M. tuberculosis* H_37_Rv strain by agar dilution method using standard drugs ethambutol, isoniazid and pyrazinamide for comparison which showed the values in the range of 3.125–50.0 µg/mL. Among the synthesized compounds, compounds **38a**, **38b**, **38c**, **38d**, **38e**, **38f** and **38g** (Fig. 7) showed potent anti-tubercular activity with MIC value (3.125 µg/mL) and comparable to standard ethambutol (MIC = 3.13 µg/mL) [43].

**Fig. 8:**
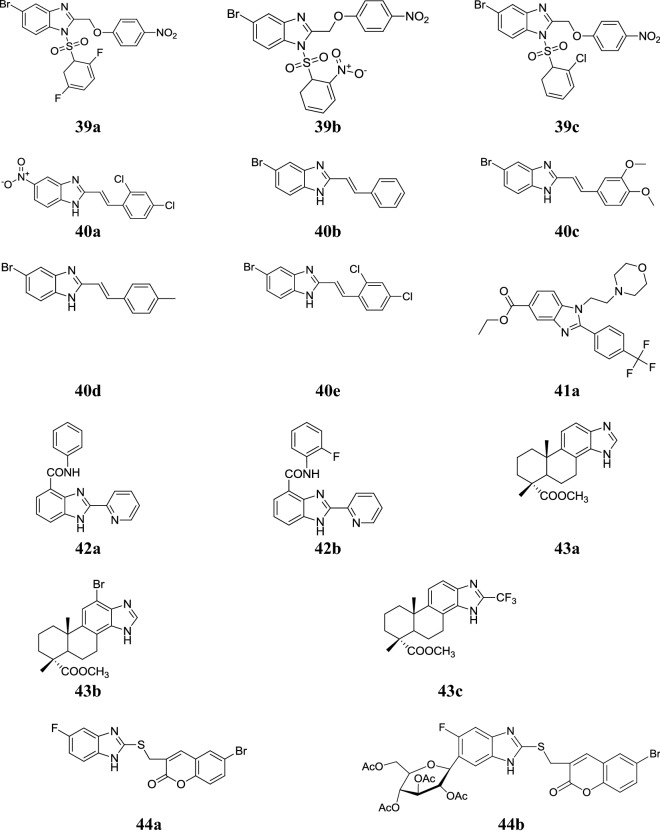
Molecular structures of compounds (**39a**–**39c**, **40a**–**40e**, **41a**, **42a**–**42b**, **43a**–**43c**, **44a**–**44b**)

Ranjith et al. developed a class of positional isomers having benzimidazole moiety and evaluated for its antitubercular potency against *M. smegmatis* (MS), *M. tuberculosis* H_37_Rv (MTB), *M. fortuitum* (MF) and MDR-TB strains using isoniazid and rifampicin as standards. Among the synthesized derivatives, compounds **39a**, **39b** and **39c** displayed significant activity against *M. tuberculosis* H_37_Rv (Table 33, Fig. 8) [44].

**Table 33:** Antitubercular screening results

Comp.	Screening results, MIC (µg/mL)			
	MTB	MS	MF	MDR-TB
**39a**	0.625	10	10	6.25
**39b**	0.625	1.25	10	6.25
**39c**	0.625	1.25	10	6.25
**Isoniazid**	0.7	50	12.5	12.5
**Rifampicin**	0.5	1.5	1.5	25

Shingalapur et al. synthesized some novel 5-(nitro/bromo)-styryl-2-benzimidazole compounds and evaluated for their in vitro anti-tubercular activity towards *M. tuberculosis* H37 Rv by alamar blue assay using streptomycin (100% inhibition) as reference. Among them, compounds **40a**, **40b**, **40c**, **40d** and **40e** showed significant antitubercular activity (Table 34, Fig. 8) [45].

**Table 34:** Antitubercular activity {MIC (µg/mL)}

Comp.	*M. tuberculosis* H37 Rv
**40a**	> 7.25 (45)
**40b**	> 7.25 (83)
**40c**	> 7.25 (54)
**40d**	> 7.25 (63)
**40e**	> 7.25 (76)

Yoon et al. prepared some new benzimidazole derivatives and evaluated for their antimycobacterial potency against *M. tuberculosis* H37Rv strain using BacTiter-Glo™ microbial cell viability (BTG) assay using six standard drugs (rifampicin, cycloserine, pyrimethamine, isoniazid, amikacin and ethambutol). In this series, compound **41a** was found to be the highly potent agent as compared to standard drugs (Table 35, Fig. 8) [46].

**Table 35:** Antimycobacterial activity of benzimidazole derivative **41a**

Comp.	*M. tuberculosis* H37Rv					
	Alamar blue			BTG		
	IC_50_ (µM)	IC_90_ (µM)	MIC (µM)	IC_50_ (µM)	IC_90_ (µM)	MIC (µM)
**41a**	16.14	44.46	100	11.52	16.53	50
**Amikacin**	0.12	0.14	0.16	0.07	0.12	0.16
**Cycloserine**	24.76	28.01	100	23.55	26.38	100
**Ethambutol**	3.45	> 200	NA	1.50	1.64	6.25
**Isoniazid**	0.19	> 5	NA	0.13	0.20	0.31
**Pyrimethamine**	25.09	28.00	100	24.27	46.37	100
**Rifampicin**	0.02	0.02	0.16	0.02	0.03	0.04

### Antiviral activity

Cheng et al. synthesized some novel benzimidazoles and demonstrated for their antiviral activity against Coxsackie virus B_3_ in VERO cells. Among the synthesized derivatives, compounds **42a** and **42b** (Fig. 8) showed potent selective activity with IC_50_ values (1.43 and 0.54 µg/mL) as compared to ribavirin (RVB) with IC_50_ value and eminent selective index (411.7 µg/mL and > 2.42) [47].

Fonseca et al. synthesized benzimidazole compounds incorporated into a hydrophenanthrene and naphthalene skeleton and screened for their in vitro antiviral activity against several RNA and DNA viruses. Among them, compounds **43a**, **43b** and **43c** (Fig. 8) displayed good activity against VZV and CMV replication and comparable to that of acyclovir and ganciclovir (Table 36) [48].

**Table 36:** Antiviral screening results of the synthesized compounds **(43a-c)**

Comp.	Antiviral potency IC_50_ (µg/mL)	
	CMV	VZV
**43a**	> 0.2	0.2–0.5
**43b**	1.1–3.2	0.6–2.8
**43c**	1.0–1.2	0.8–1.4
**Acyclovir**	–	0.3–3.0
**Ganciclovir**	0.9–1.5	–

Hwu et al. developed some new benzimidazole derivatives bearing coumarin ring and evaluated for their antiviral activity against the hepatitis C virus. Among the synthesized derivatives, compounds **44a** and **44b** (Fig. 8) were found to be most active and showed EC_50_ values (3.4 µM and 4.1 µM) [49].

**Fig. 9:**
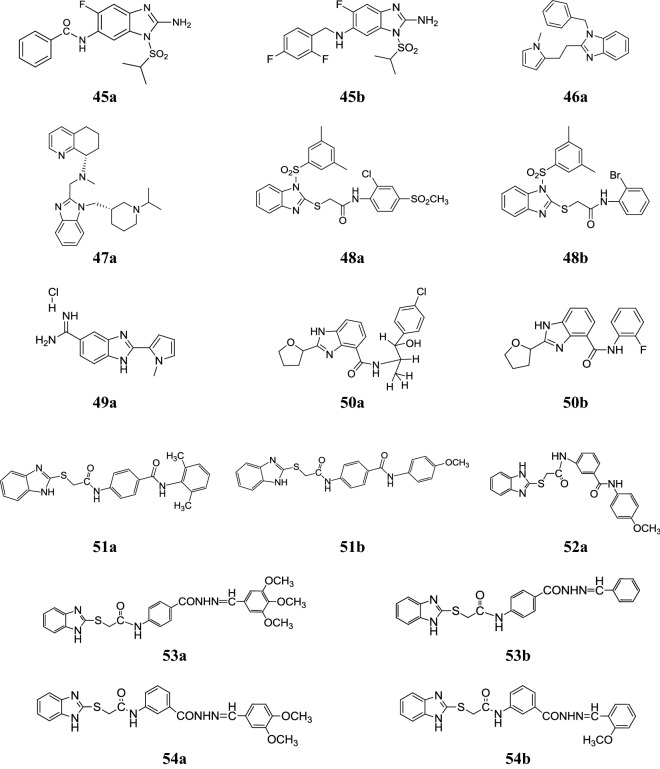
Molecular structures of compounds (**45a**–**45b**, **46a**, **47a**, **48a**–**48b**, **49a**, **50a**–**50b**, **51a**–**51b**, **52a**, **53a**–**53b**, **54a**–**54b**)

Li et al. synthesized a class of novel benzimidazoles and assessed for their hepatitis B virus inhibition activity. Among them, compounds **45a** and **45b** showed outstanding anti-HBV potency and comparable to lamivudine and adefovir (Table 37, Fig. 9) [50].

**Table 37:** Antiviral activity results of the synthesized compounds (**45a**–**45b**)

Comp.	IC_50_ (µM)	CC_50_ (µM)	SI
**45a**	0.70	192	274
**45b**	0.70	86	123
**Lamivudine**	0.38	> 1000	> 2632
**Adefovir**	1.7	57	34

Luo et al. developed few novel benzimidazole compounds and evaluated for their anti-hepatitis B virus (HBV) activity and cytotoxicity in HepG 2.2.15 cells. In this study, compound **46a** showed significant antiviral activity using lamivudine as reference (Table 38, Fig. 9) [51].

**Table 38:** Antiviral activity results of the synthesized compounds **46a**

Comp.	IC_50_ (µM)	CC_50_ (µM)	SI
**46a**	< 0.41	33.3	81.2
**Lamivudine**	5	0.16	3.13

Miller et al. designed a series of *N*-substituted benzimidazoles as CXCR4 antagonists. In this series, compound **47a** (Fig. 9) exhibited promising antiviral activity having IC_50_ of 2 nM, a 1000-fold cytotoxicity window and a twofold protein shift. A modification in side chain and stereochemical optimization led to significantly enhancement in potency and protein shift to afford compounds with low nanomolar anti-HIV activity [52].

Monforte et al. synthesized some novel *N*_1_-aryl-2-arylthioacetamido-benzimidazoles and screened for their human immunodeficiency virus type-1 (HIV-1) inhibitor activity. In this series, compounds **48a** and **48b** were found as the most active compounds with no toxicity (Table 39, Fig. 9) [11].

**Table 39:** Anti-RT and anti-HIV-1 activities, cytotoxicity and selectivity index in MT-4 cells

Comp.	IC_50_ (µM)	EC_50_ (µM)	CC_50_ (µM)	SI
**48a**	0.12 ± 0.035	0.04 ± 0.01	> 221.59	> 5540
**48b**	0.18 ± 0.018	0.06 ± 0.02	≥ 235.64	≥ 3927
**Nevirapine**	2.55 ± 0.93	0.19 ± 0.06	> 15.02	> 79
**Efavirenz**	0.032 ± 0.009	0.006 ± 0.0001	> 1056	> 6.34

Starcevic et al. synthesized 2-substituted-5-amidino-benzimidazoles and assessed for their in vitro inhibitory activity against GMK cell line and HeLa cell line by MTT assay. From this series, compound **49a** showed prominent activity against all four types of viruses with no cytotoxicity (Table 40, Fig. 9) [12].

**Table 40:** Antiviral activity EC_50_ (µM)

Comp.	HeLa		GMK	
	Adenovirus 5	Herpesvirus 1	Coxsackievirus B5	Echovirus 7
**49a**	5.9	30	3.5	5

Zhang et al. reported some new benzimidazole derivatives and screened for their anti-Coxsackie virus B3 (CVB3) activity in VERO cells. In this series, compounds **50a** and **50b** (Fig. 9) exhibited better inhibitory activity with IC_50_ values (5.30 and 1.06 µg/mL) together with good selective indexes (12.1 and 7.5) than those of ribavirin (RBV) with IC_50_ value 353.33 [53].

### Anticancer activity

In this study, Tahlan et al. developed a new class of benzimidazole benzamide compounds and demonstrated for its anticancer activity against cancer cell line (HCT116) by SRB method and compared to standard drugs (5-fluorouracil). From the synthesized derivatives, compound **51a** and **51b** (Fig. 9) showed the significant anticancer activity (Table 41) [3].

**Table 41:** Anticancer activity results of synthesized compounds (**51a** and **51b**)

Comp.	Cancer cell line (IC_50_ = μM)
	HCT116
**51a**	5.85
**51b**	4.53
**5-Fluorouracil**	9.99

Designed and synthesized a novel series of benzimidazole derivatives by Tahlan et al. and evaluated for its anticancer potency towards cancer cell line (HCT116) by SRB assay. In this series, compound **52a** (Fig. 9) was found to be most promising anticancer compound. The significant result of the most active compound is shown in Table 42 [2].

**Table 42:** Anticancer activity results of synthesized compound (**52a**)

Comp.	Cancer cell line (IC_50_ = μM)
	HCT116
**52a**	4.12
**5-Fluorouracil**	7.69

### Antimicrobial activity

Novel class of benzimidazole Schiff base derivatives has been synthesized by Tahlan et al. and evaluated for their antimicrobial activity against Gram positive and Gram negative bacterial and fungal species by tube dilution method. In this series, compounds **53a** and **53b** (Fig. 9) displayed potent antifungal activity against *A. niger* and *C. albicans*. The significant result of the active compounds is shown in Table 43 [54].

**Table 43:** Antimicrobial results of compounds (**53a**–**53b**)

Comp.	Microbial strains (MIC = µM/mL)						
	Bacterial strains					Fungal strains	
	*S. aureus*	*E. coli*	*B. subtilis*	*P. aeruginosa*	*S. enterica*	*C. albicans*	*A. niger*
**53a**	9.62	9.62	2.41	2.41	4.81	2.41	1.20
**53b**	5.82	2.91	5.82	5.82	5.82	1.46	2.91
**Cefadroxil**	1.72	1.72	1.72	1.72	1.72	–	–
**Fluconazole**	–	–	–	–	–	2.04	2.04

Tahlan et al. synthesized a class of benzimidazole Schiff base derivatives and screened for its antimicrobial activity toward selected microbial species. From the series compounds **54a** and **54b** (Fig. 9) exhibited promising antimicrobial activity towards bacterial and fungal species. The significant result of the active compounds is shown in Table 44 [55].

**Table 44:** Antimicrobial results of compounds (**54a**–**54b**)

Comp.	Microbial strains (MIC = µM/mL)						
	Bacterial species					Fungal species	
	*B. subtilis*	*P. aeruginosa*	*E. coli*	*S. typhi*	*K. pneumoniae*	*C. albicans*	*A. niger*
**54a**	1.28	1.28	1.28	2.55	5.11	5.11	2.55
**54b**	0.68	0.68	2.72	2.72	5.44	5.44	2.72
**Cefadroxil**	1.73	3.46	3.46	0.86	3.46	–	–
**Fluconazole**	–	–	–	–	–	4.08	4.08

## Conclusion

The present review based on reported heterocyclic benzimidazole derivatives which displayed the significant biological potentials in medicinal chemistry. Benzimidazole moiety is the key building block for several heterocyclic scaffolds that play central role in the biologically functioning of essential molecules and are surprisingly effective with their restraint movement and favorable selectiveness. The present review article is based on various reported pharmacological activities of heterocyclic 1*H*-benzimidazole derivatives. The review article shows the pharmacological activities of the reported synthesized benzimidazole derivatives in medicinal field. We hope this paper may be helpful in the development of new derivatives of benzimidazole based on medicinal chemistry and as well as designing of new drug molecule in future.

## Data Availability

Not applicable

## Abbreviations

AChE: acetylcholinesterase
AD: Alzheimer’s disease
TB: tuberculosis
*M. tuberculosis*: *Mycobacterium tuberculosis*
GI: *Giardia intestinalis*
TV: *Trichomonas vaginalis*
EH: *Entamoeba histolytica*
LM: *Leishmania mexicana*
ABZ: albendazole
SI: selectivity index
TS: *Trichinella spiralis*
TBZ: triclabendazole
MBZ: mebendazole
EWG: electron withdrawing groups
VZV: varicella-zoster virus
CMV: cytomegalovirus
WHO: World Health Organization
CRC: colorectal tumour
CSC: cancer stem cell
CDK: cyclin-dependent kinase

## References

[1] Alaqeel SI (2017). Synthetic approaches to benzimidazoles from o-phenylenediamine: a literature review. J Saudi Chem Soc.

[2] Tahlan S, Ramasamy K, Lim SM, Shah SAA, Mani V, Narasimhan B (2019). Design, synthesis and therapeutic potential of 3-(2-(1*H*-benzo[*d*]imidazol-2-ylthio) acetamido)-*N*-(substituted phenyl)benzamide analogues. Chem Cent J.

[3] Tahlan S, Ramasamy K, Lim SM, Shah SAA, Mani V, Narasimhan B (2019). (2-(1*H*-Benzo[*d*]imidazol-2-ylthio)acetamido)-*N*-(substituted phenyl) benzamides: design, synthesis and biological evaluation. BMC Chem.

[4] Alpan AS, Parlar S, Carlino L, Tarikogullari AH, Alptuzun V, Gunes HS (2013). Synthesis biological activity and molecular modeling studies on 1*H*-benzimidazole derivatives as acetylcholinesterase inhibitors. Bioorg Med Chem.

[5] Andrzejewskaa M, Yepez-Mulia L, Tapia A, Cedillo-Rivera R, Laudy AE, Starosciak BJ, Kazimierczuk Z (2004). Synthesis and antiprotozoal and antibacterial activities of S-substituted 4,6-dibromo- and 4,6-dichloro-2-mercaptobenzimidazoles. Eur J Pharm Sci.

[6] Achar KCS, Hosamani KM, Seetharamareddy HR (2010). *In*-*vivo* analgesic and anti-inflammatory activities of newly synthesized benzimidazole derivatives. Eur J Med Chem.

[7] Gaba M, Gaba P, Uppal D, Dhingra N, Bahiad MS, Silakari O, Mohan C (2015). Benzimidazole derivatives: search for GI-friendly anti-inflammatory analgesic agents. Acta Pharm Sin B.

[8] Wang XJ, Xi MY, Fu JH, Zhang FR, Cheng GF, Yin DL, You QD (2012). Synthesis, biological evaluation and SAR studies of benzimidazole derivatives as H_1_-antihistamine agents. Chin Chem Lett.

[9] Camacho J, Barazarte A, Gamboa N, Rodrigues J, Rojas R, Vaisberg A, Gilman R, Charris J (2011). Synthesis and biological evaluation of benzimidazole-5-carbohydrazide derivatives as antimalarial, cytotoxic and antitubercular agents. Bioorg Med Chem.

[10] Desai NC, Shihory NR, Kotadiya GM, Desai P (2014). Synthesis, antibacterial and antitubercular activities of benzimidazole bearing substituted 2-pyridone motifs. Eur J Med Chem.

[11] Monforte AM, Ferro S, Luca LD, Surdo GL, Morreale F, Pannecouque C, Balzarini J, Chimirri A (2014). Design and synthesis of *N*_1_-aryl-benzimidazoles 2-substituted as novel HIV-1 non-nucleoside reverse transcriptase inhibitors. Bioorg Med Chem.

[12] Starcevic K, Kralj M, Ester K, Sabol I, Grce M, Pavelic K, Karminski-Zamolaa G (2007). Synthesis, antiviral and antitumor activity of 2-substituted-5-amidino-benzimidazoles. Bioorg Med Chem.

[13] Salahuddin Shaharyar M, Mazumder A (2017). Benzimidazoles: a biologically active compounds. Arab J Chem.

[14] Hernandez-Luis F, Hernandez-Campos A, Castillo R, Navarrete-Vazquez G, Soria-Arteche O, Hernandez-Hernandez M, Yepez-Mulia L (2010). Synthesis and biological activity of 2-(trifluoromethyl)-1*H*-benzimidazole derivatives against some protozoa and *Trichinella spiralis*. Eur J Med Chem.

[15] Bandyopadhyay P, Sathe M, Tikar SN, Yadav R, Sharma P, Kumar A, Kaushik MP (2014). Synthesis of some novel phosphorylated and thiophosphorylated benzimidazoles and benzothiazoles and their evaluation for larvicidal potential to *Aedes albopictus* and *Culex quinquefasciatus*. Bioorg Med Chem Lett.

[16] Gong Y, Karakaya SS, Guo X, Zheng P, Gold B, Ma Y, Little D, Roberts J, Warrier T, Jiang X, Pingle M, Nathan CF, Liu G (2014). Benzimidazole-based compounds kill *Mycobacterium tuberculosis*. Eur J Med Chem.

[17] Dai D, Burgeson JR, Gharaibeh DN, Moore AL, Larson RA, Cerruti NR, Amberg SM, Bolken TC, Hruby DE (2013). Discovery and optimization of potent broad-spectrum arenavirus inhibitors derived from benzimidazole. Bioorg Med Chem Lett.

[18] Yoon YK, Ali MA, Wei AC, Choon TS, Khaw KY, Murugaiyah V, Osman H, Masand VH (2013). Synthesis characterization and molecular docking analysis of novel benzimidazole derivatives as cholinesterase inhibitors. Bioorg Chem.

[19] DIaz-Chiguer DL, Marquez-Navarro A, Nogueda-Torres B, Leon-Avila GL, Perez-Villanueva J, Hernandez-Campos A, Castillo R, Ambrosio JR, Nieto-Meneses R, Yepez-Mulia L, Hernandez-Luis F (2012). In vitro and in vivo trypanocidal activity of some benzimidazole derivatives against two strains of *Trypanosoma cruzi*. Acta Trop.

[20] Hernandez-Covarrubias C, Vilchis-Reyes MA, Yepez-Mulia L, Sanchez-Diaz R, Navarrete-Vazquez G, Hernandez-Campos A, Castillo R, Hernandez-Luis F (2012). Exploring the interplay of physicochemical properties, membrane permeability and giardicidal activity of some benzimidazole derivatives. Eur J Med Chem.

[21] Kopanska K, Najda A, Zebrowska J, Chomicz L, Piekarczyk J, Myjakd P, Bretnera M (2004). Synthesis and activity of 1*H*-benzimidazole and 1*H*-benzotriazole derivatives as inhibitors of *Acanthamoeba castellanii*. Bioorg Med Chem.

[22] Mavrova AT, Vuchev D, Anichina K, Vassilev N (2010). Synthesis, antitrichinnellosis and antiprotozoal activity of some novel thieno[2,3-*d*]pyrimidin-4(3*H*)-ones containing benzimidazole ring. Eur J Med Chem.

[23] Navarrete-Vazquez G, Rojano-Vilchis MM, Yepez-Mulia L, Melendez V, Gerena L, Hernandez-Campos A, Castillo R, Hernandez-Luis F (2006). Synthesis and antiprotozoal activity of some 2-(trifluoromethyl)-1*H*-benzimidazole bioisosteres. Eur J Med Chem.

[24] Marquez-Navarro A, Nogueda-Torres B, Hernandez-Campos A, Soria-Arteche O, Castillo R, Rodriguez-Morales S, Yepez-Mulia L, Hernandez-Luis F (2009). Anthelmintic activity of benzimidazole derivatives against *Toxocara canis* second-stage larvae and *Hymenolepis nana* adults. Acta Trop.

[25] Oh S, Kim S, Kong S, Yang G, Lee N, Han D, Goo J, Siqueira-Neto JL, Freitas-Junior LH, Song R (2014). Synthesis and biological evaluation of 2,3-dihydroimidazo[1,2-*a*] benzimidazole derivatives against *Leishmania donovani* and *Trypanosoma cruzi*. Eur J Med Chem.

[26] Palomares-Alonso F, Jung-Cook H, Perez-Villanueva J, Piliado JC, Rodriguez-Morales S, Palencia-Hernandez G, Lopez-Balbiaux N, Hernandez-Campos A, Castillo R, Hernandez-Luis F (2009). Synthesis and in vitro cysticidal activity of new benzimidazole derivatives. Eur J Med Chem.

[27] Perez-Villanueva J, Hernandez-Campos A, Yepez-Mulia L, Mendez-Cuesta C, Mendez-Lucio O, Hernandez-Luis F, Castillo R (2013). Synthesis and antiprotozoal activity of novel 2-{[2-(1*H*-imidazol-1-yl)ethyl]sulfanyl}-1*H*-benzimidazole derivatives. Bioorg Med Chem Lett.

[28] Sondhi SM, Rajvanshi S, Johar M, Bharti N, Azam A, Singh AK (2002). Anti-inflammatory, analgesic and antiamoebic activity evaluation of pyrimido[1,6-*a*] benzimidazole derivatives synthesized by the reaction of ketoisothiocyanates with mono and diamines. Eur J Med Chem.

[29] Torres-Gomez H, Hernandez-Nunez E, Leon-Rivera I, Guerrero-Alvarez J, Cedillo-Rivera R, Moo-Puc R, Argotte-Ramos R, Rodriguez-Gutierrez MC, Chan-Bacab MJ, Navarrete-Vazquez G (2008). Design, synthesis and in vitro antiprotozoal activity of benzimidazolepentamidine hybrids. Bioorg Med Chem Lett.

[30] Velazquez-Lopez JM, Hernandez-Campos A, Yepez-Mulia L, Tellez-Valencia A, Flores-Carillo P, Nieto-Meneses R, Castillo R (2016). Synthesis and trypanocidal activity of novel benzimidazole derivatives. Bioorg Med Chem Lett.

[31] El-Feky SA, Thabet HK, Ubeid MT (2014). Synthesis, molecular modeling and anti-inflammatory screening of novel fluorinated quinoline incorporated benzimidazole derivatives using the Pfitzinger reaction. J Fluorine Chem.

[32] Jesudason EP, Sridhar SK, Malar EJP, Shanmugapandiyan P, Inayathullah M, Arul V, Selvaraj D, Jayakumar R (2009). Synthesis, pharmacological screening, quantum chemical and in vitro permeability studies of *N*-Mannich bases of benzimidazoles through bovine cornea. Eur J Med Chem.

[33] Mariappan G, Hazarika R, Alam F, Karki R, Patangia U, Nath S (2015). Synthesis and biological evaluation of 2-substituted benzimidazole derivatives. Arab J Chem.

[34] Paramashivappa R, Kumar PP, Rao PVS, Rao AS (2003). Design, synthesis and biological evaluation of benzimidazole/benzothiazole and benzoxazole derivatives as cyclooxygenase inhibitors. Bioorg Med Chem Lett.

[35] Ravindernath A, Reddy MS (2017). Synthesis and evaluation of anti-inflammatory, antioxidant and antimicrobial activities of densely functionalized novel benzo[*d*] imidazolyl tetrahydropyridine carboxylates. Arab J Chem.

[36] Sondhi SM, Singh N, Kumar A, Lozach O, Meijer L (2006). Synthesis, anti-inflammatory, analgesic and kinase (CDK-1, CDK-5 and GSK-3) inhibition activity evaluation of benzimidazole/benzoxazole derivatives and some Schiff’s bases. Bioorg Med Chem.

[37] Vicini P, Incerti M, Amoretti L, Ballabeni V, Tognolini M, Barocelli E (2002). Synthesis and pharmacological properties of benzisothiazole/benzimidazole derivatives with acidic groups. Farmaco.

[38] Yang H, Murigi FN, Wang Z, Li J, Jin H, Tu Z (2015). Synthesis and in vitro characterization of cinnoline and benzimidazole analogues as phosphodiesterase 10A inhibitors. Bioorg Med Chem Lett.

[39] Divatia SM, Rajani DP, Rajani SD, Patel HD (2014). Novel thiosemicarbazone derivatives containing benzimidazole moiety: green synthesis and anti-malarial activity. Arab J Chem.

[40] Toro P, Klahn AH, Pradines B, Lahoz F, Pascual A, Biot C, Arancibia R (2013). Organometallic benzimidazoles: synthesis, characterization and antimalarial activity. Inorg Chem Commun.

[41] Kalalbandi VKA, Seetharamappa J, Katrahalli U, Bhat KG (2014). Synthesis, crystal studies, anti-tuberculosis and cytotoxic studies of 1-[(2*E*)-3-phenylprop-2-enoyl]-1*H*-benzimidazole derivatives. Eur J Med Chem.

[42] Park B, Awasthi D, Chowdhury SR, Melief EH, Kumar K, Knudson SE, Slayden RA, Ojima I (2014). Design, synthesis and evaluation of novel 2,5,6-trisubstituted benzimidazoles targeting FtsZ as antitubercular agents. Bioorg Med Chem.

[43] Ramprasad J, Nayak N, Dalimba U, Yogeeswari P, Sriram D, Peethambar SK, Achur R, Kumar HSS (2015). Synthesis and biological evaluation of new imidazo[2,1-*b*][1,3,4] thiadiazole-benzimidazole derivatives. Eur J Med Chem.

[44] Ranjith PK, Rajeesh P, Haridas KR, Susanta NK, Row TNG, Rishikesan R, Kumari NS (2013). Design and synthesis of positional isomers of 5 and 6-bromo-1-[(phenyl)sulfonyl]-2-[(4-nitrophenoxy)methyl]-1*H* benzimidazoles as possible antimicrobial and antitubercular agents. Bioorg Med Chem Lett.

[45] Shingalapur RV, Hosamani KM, Keri RS (2009). Synthesis and evaluation of in vitro anti-microbial and anti-tubercular activity of 2-styryl benzimidazoles. Eur J Med Chem.

[46] Yoon YK, Ali MA, Wei AC, Choon TS, Ismail R (2015). Synthesis and evaluation of antimycobacterial activity of new benzimidazole aminoesters. Eur J Med Chem.

[47] Cheng J, Xie J, Luo X (2005). Synthesis and antiviral activity against Coxsackie virus B3 of some novel benzimidazole derivatives. Bioorg Med Chem Lett.

[48] Fonseca T, Gigante B, Marques MM, Gilchrist TL, Clercq ED (2004). Synthesis and antiviral evaluation of benzimidazoles, quinoxalines and indoles from dehydroabietic acid. Bioorg Med Chem.

[49] Hwu JR, Singha R, Hong SC, Chang YH, Das AR, Vliegen I, Clercq ED, Neyts J (2008). Synthesis of new benzimidazole–coumarin conjugates as anti-hepatitis C virus agents. Antivir Res.

[50] Li YF, Wang GF, Luo Y, Huang WG, Tang W, Feng CL, Shi LP, Ren YD, Zuo JP, Lu W (2007). Identification of 1-isopropylsulfonyl-2-amine benzimidazoles as a new class of inhibitors of hepatitis B virus. Eur J Med Chem.

[51] Luo Y, Yao JP, Yang L, Feng CL, Tang W, Wang GF, Zuo JP, Lu W (2010). Design and synthesis of novel benzimidazole derivatives as inhibitors of hepatitis B virus. Bioorg Med Chem.

[52] Miller JF, Turner EM, Gudmundsson KS, Jenkinson S, Spaltenstein A, Thomson M, Wheelan P (2010). Novel N-substituted benzimidazole CXCR56 antagonists as potential anti-HIV agents. Bioorg Med Chem Lett.

[53] Zhang ZL, Sun ZJ, Xue F, Luo XJ, Xiu NY, Teng L, Peng ZG (2009). Design, synthesis and biological activity of some novel benzimidazole derivatives against Coxsackie virus B_3_. Chin Chem Lett.

[54] Tahlan S, Narasimhan B, Lim SM, Ramasamy K, Mani V, Shah SAA (2018). Mercaptobenzimidazole Schiff bases: design, synthesis, antimicrobial studies and anticancer activity on HCT-116 cell line. Mini-Rev Med Chem.

[55] Tahlan S, Narasimhan B, Lim SM, Ramasamy K, Mani V, Shah SAA (2018). Design, synthesis, SAR study, antimicrobial and anticancer evaluation of novel -mercaptobenzimidazole azomethine derivatives. Mini-Rev Med Chem.

